# Reduction of a nymphal instar in a dampwood termite: heterochronic shift in the caste differentiation pathways

**DOI:** 10.1186/s13227-019-0123-8

**Published:** 2019-05-16

**Authors:** Ryotaro Nii, Kohei Oguchi, Junpei Shinji, Shigeyuki Koshikawa, Toru Miura

**Affiliations:** 1 Graduate School of Environmental Science, Hokkaido University, Sapporo, Hokkaido 060-0810 Japan; 2 Misaki Marine Biological Station, School of Science, The University of Tokyo, Miura, Kanagawa 238-0225 Japan; 3 Faculty of Environmental Earth Science, Hokkaido University, Sapporo, Hokkaido 060-0810 Japan

**Keywords:** Termites, Alate differentiation, Caste differentiation, Nymphal instar, Wing formation, Gene expression, Heterochrony

## Abstract

**Background:**

Generally in termites, alates differentiate through multiple nymphal instars which gradually develop wing buds. However, in a dampwood termite, *Hodotermopsis sjostedti*, alates molt directly from a single nymphal instar with short wing buds. In this study, to examine the mechanism underlying the wing formation during the alate differentiation in *H. sjostedti*, histological and morphological observations were carried out on the developmental process of wing formation during the nymphal instar, in comparison with those in *Zootermopsis nevadensis*, which has two nymphal instars. Furthermore, the expression patterns of genes that are thought to be responsible for wing formation, i.e., wing-patterning genes and genes encoding hormone-related factors, were quantified during alate differentiation and compared between the two species.

**Results:**

The results showed that, in *H. sjostedti*, wings were formed in a complicatedly folded shape, not only inside the wing buds as seen in *Z. nevadensis*, but also under the dorsal thoracic cuticle, where the wing tips shifted toward the median thoracic part. Accordingly, the wing expansion pattern also differed from that in *Z. nevadensis*. Furthermore, the results of real-time qRT-PCR on overall expression profiles of wing-patterning genes and hormone-related genes suggest that the single nymphal instar in *H. sjostedti* well resembles to the second nymphal instar in *Z. nevadensis*. In particular, significant upregulation of *vestigial* (*vg*) and downregulation of *Krüppel homolog 1* (*Kr*-*h1*) that were observed at the second nymphal instar in *Z. nevadensis* apparently occurred during the single nymphal instar in *H. sjostedti*.

**Conclusion:**

The developmental events for wing formation are compacted into a single nymphal instar in *H. sjostedti*, and as a result, the unique wing formation is seen to compensate for the spatial restriction inside small wing buds, leading to the completion of functional wings.

**Electronic supplementary material:**

The online version of this article (10.1186/s13227-019-0123-8) contains supplementary material, which is available to authorized users.

## Background

**Fig. 1:**
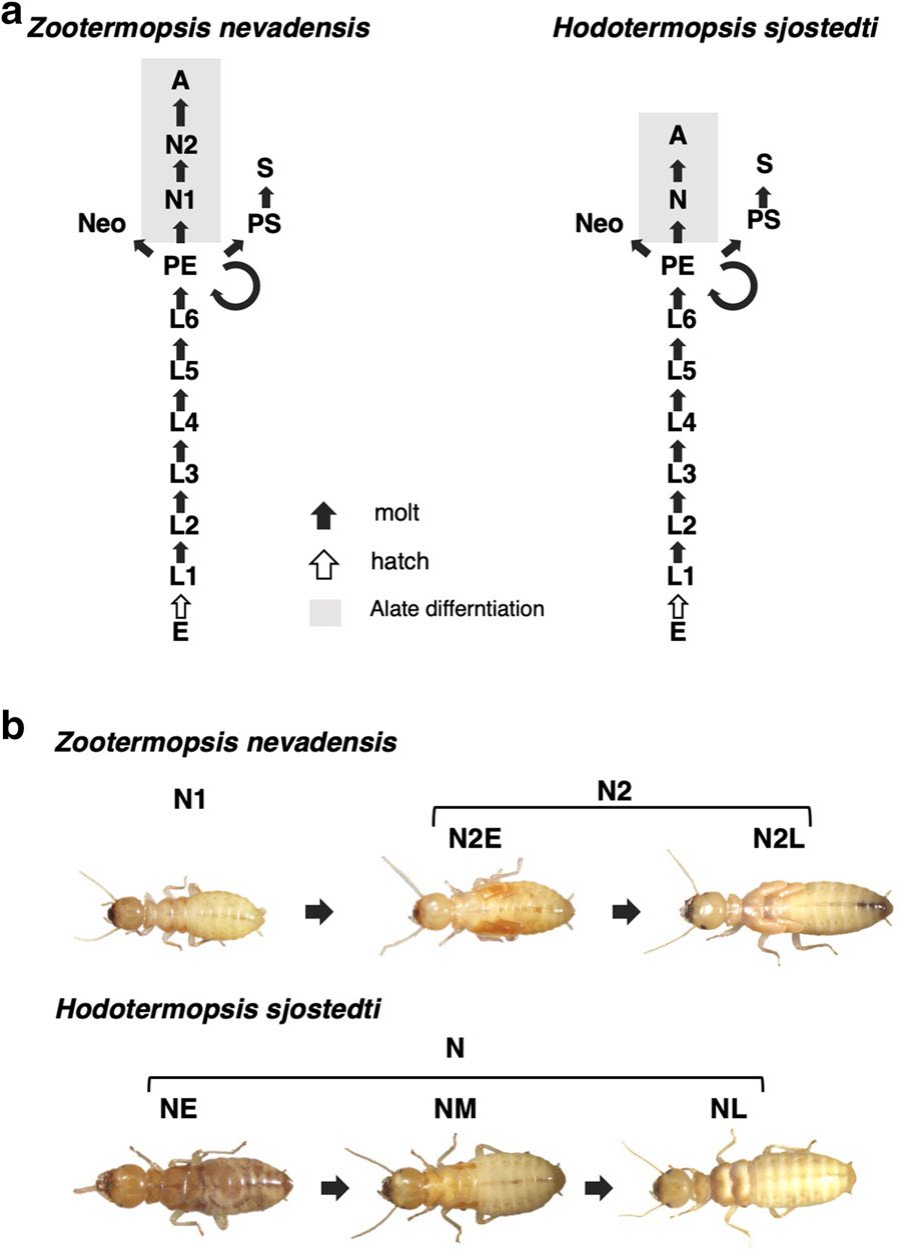
**a** Caste differentiation pathways in the two termite species studied here: *Zootermopsis nevadensis* (left) and *Hodotermopsis sjostedti* (right). Abbreviations are: E, egg; L, larva, i.e., immatures without wing buds; PE, pseudergate; N, nymph, i.e., immatures with wing buds/pads; A, alate; Neo, neotenic; PS, presoldier; S, soldier. **b** Nymphal stages of the two species that were focused on in this study. In this study, the last nymphal instar was divided into several stages in order to understand the developmental processes. Abbreviations are: N1, first-instar nymph; N2E, early second-instar nymph; and N2L, late second-instar nymph in *Z. nevadensis*, and NE, early nymph; NM, mid-nymph, and NL, late nymph in *H. sjostedti*

Eusocial insects such as bees, wasps, ants and termites show highly organized sociality with divisions of labor among morphologically and behaviorally differentiated castes [1]. Patterns of caste differentiation in termites differ from those in social hymenopterans, mostly due to the modes of postembryonic development: hemimetabolous in termites and holometabolous in hymenopterans [2]. In termites, the number of instars can vary among species and even larval (immatures without wing buds) and nymphal instars (immatures with wing buds) can perform colony tasks, so that soldiers and workers possess immature characteristics [3]. Therefore, the caste differentiation pathways are diverse among termite lineages, suggesting that the pattern of postembryonic development, i.e., molting and morphogenesis, has evolved in association with social evolution (Fig. 1).

The caste differentiation pathways can be classified into two major patterns depending on the timing of differentiation into reproductive individuals (imagos): linear and forked pathways [3–5]. In the forked pathways, alate development proceeds exclusively via nymphal instars, while worker and soldier development proceed via distinct larval instars. In contrast, in the linear pathways, all individuals go through larval instars without wing buds up to late instars (i.e., pseudergates that perform worker tasks but still possess potential to develop into alates). A fraction of these pseudergates differentiate into alates through nymphal instars with apparent wing buds or wing pads [4, 6, 7]. The forked pathways are seen in Mastotermitidae, Hodotermitidae, and most species of Rhinotermitidae and Termitidae, while the linear pathways are seen in Archotermopsidae, Kalotermitidae, Serritermitidae and a part of Rhinotermitidae [3, 8].

In termites, although the number of moltings that are required for the soldier differentiation is constant among species (2 moltings via presoldier), the number of nymphal instars is diverse among termite lineages [4, 6, 7]. Generally, termites possess multiple nymphal instars through which wing buds are gradually developed, forming complete wings [3]. For example, in Termitidae showing the forked pathways, there are 6 nymphal instars [9]. Species belonging to Archotermopsidae, which are thought to have primitive characteristics, possess 2 or more successive nymphal instars [4, 10].

However, in the Japanese dampwood termite *Hodotermopsis sjostedti*, which belongs to Archotermopsidae, is distributed in parts of East and Southeast Asia, pseudergates differentiate into alates through only a single nymphal instar with short wing buds (Fig. 1, [11, 12]). Other related species such as *Zootermopsis nevadensis* (Archotermopsidae) possess 2 nymphal instars, i.e., short and long wing-budded nymphs (Fig. 1, [10]). In these species, wing buds gradually develop and alate wings form inside the long wing buds (wing pads) at the final nymphal instar. This suggests that the developmental process of alate differentiation in *H. sjostedti* is distinctive, since wings should be formed inside the small wing buds of the first-instar nymphs.

In this study, therefore, to clarify the mechanism underlying the wing formation during the alate differentiation in *H. sjostedti*, histological and morphological observations were carried out on the developmental process of wing formation during its nymphal instar, in comparison with those in *Z. nevadensis*. Furthermore, expression patterns of genes that are thought to be responsible for the wing formation, i.e., wing-patterning genes and genes for hormone-related factors, were quantified during the course of alate development and compared between the two species. The regulatory genes involved in wing formation are known in several insects (e.g., [13–15]). In addition, hormone-related factors involved in molting and metamorphosis have also been reported in many insects (e.g., [16–20]). Altogether, 5 wing-patterning genes and 9 hormone-related genes were identified, and their expression patterns were analyzed during alate differentiation in the two focal species. Results clearly showed that the overall expression patterns at the second nymphal instar in *Z. nevadensis* apparently shifted to the single nymphal instar in *H. sjostedti*.

## Results

### Histological observations during wing formation

**Fig. 2:**
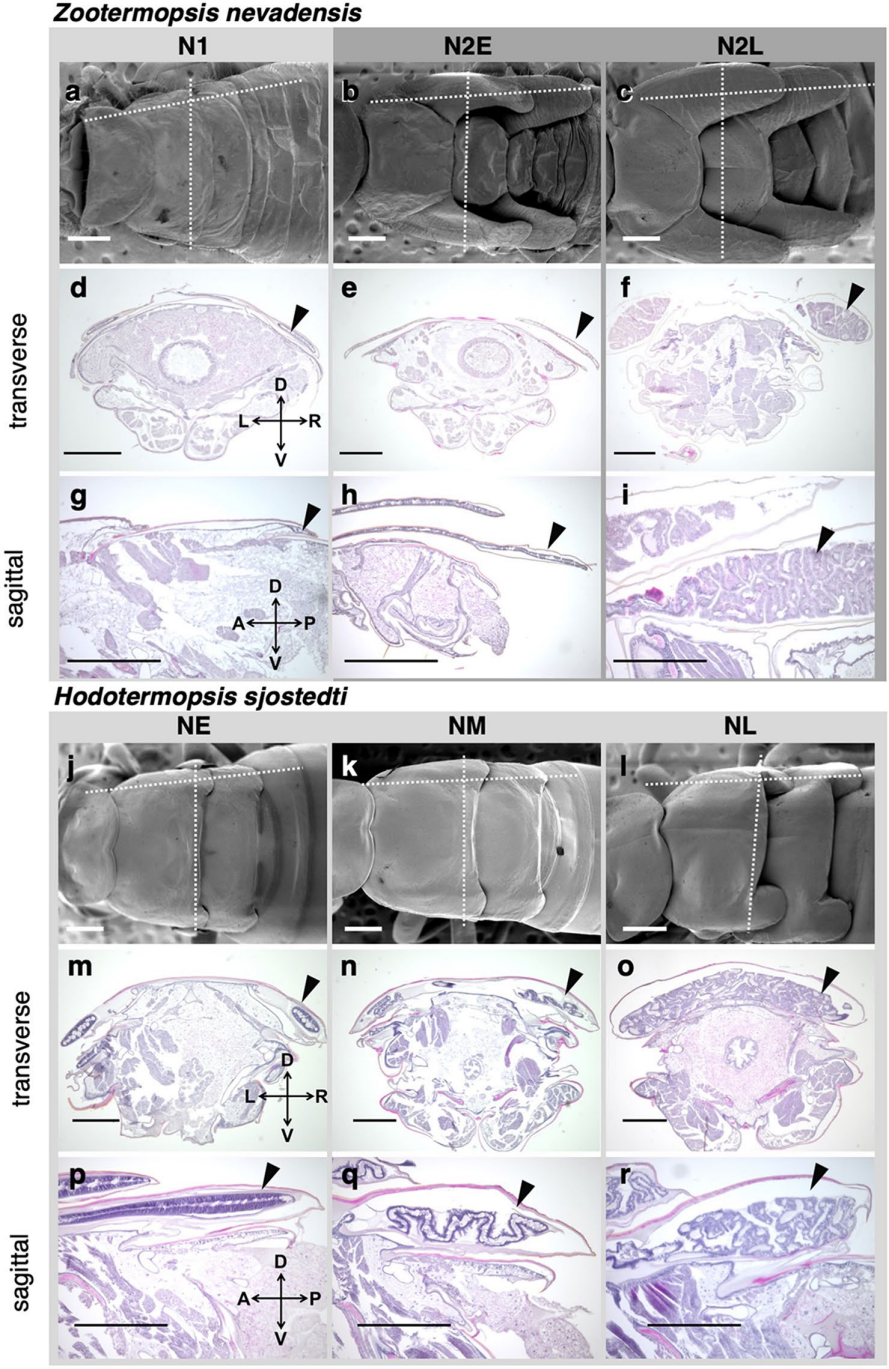
Morphological and histological transitions during nymphal instars in *Z. nevadensis* (**a**–**i**) and *H. sjostedti* (**j**–**r**). Developmental processes of nymphal instars are divided into three stages: N1 (first-instar nymph; **a**, **d**, **g**), N2E (early second-instar nymph; **b**, **e**, **h**) and N2L (late second-instar nymph; **c**, **f**, **i**) in *Z. nevadensis*; and NE (early nymph; **j**, **m**, **p**), NM (mid-nymph; **k**, **n**, **q**), and NL (late nymph; **l**, **o**, **r**) in *H. sjostedti*. Arrowheads indicate epithelial tissues of developing wings. All scale bars indicate 500 µm

Based on histological observations on paraffin sections of thoracic parts of nymphs, the developmental patterns of wings inside wing buds were compared between *H. sjostedti* and *Z. nevadensis* (Fig. 2). In *Z. nevadensis*, short wing buds of the first-instar nymphs (N1) elongated to become long wing buds through a molt into the second-instar nymphs (N2) (Fig. 2a–c). Inside the wing buds, a thin layer of epithelial tissue was seen (Fig. 2d, e, g, h). Prior to the molt into alates, epithelial tissues proliferated extensively and the layer was folded together with the newly formed cuticular layer inside the wing buds (Fig. 2f, i).

In the case of *H. sjostedti*, at the early stage of nymphs (NE), a single layer of epithelial tissue was observed under the cuticle of short wing buds (Fig. 2m, p). Later, at the mid-stage of nymphs (NM), the epithelial layer started to proliferate, forming a folded structure (Fig. 2n, q). Then, at the late stage of nymphs (NL), the folded structure became thicker and more complicated (Fig. 2o, r). The proliferated epithelial tissues that would form wings could not be contained inside the wing buds. So, as a result, the tissues of folded epithelial layers were seen in the median region of thoracic parts (Fig. 2o).

### SEM observations on wing expansion

**Fig. 3:**
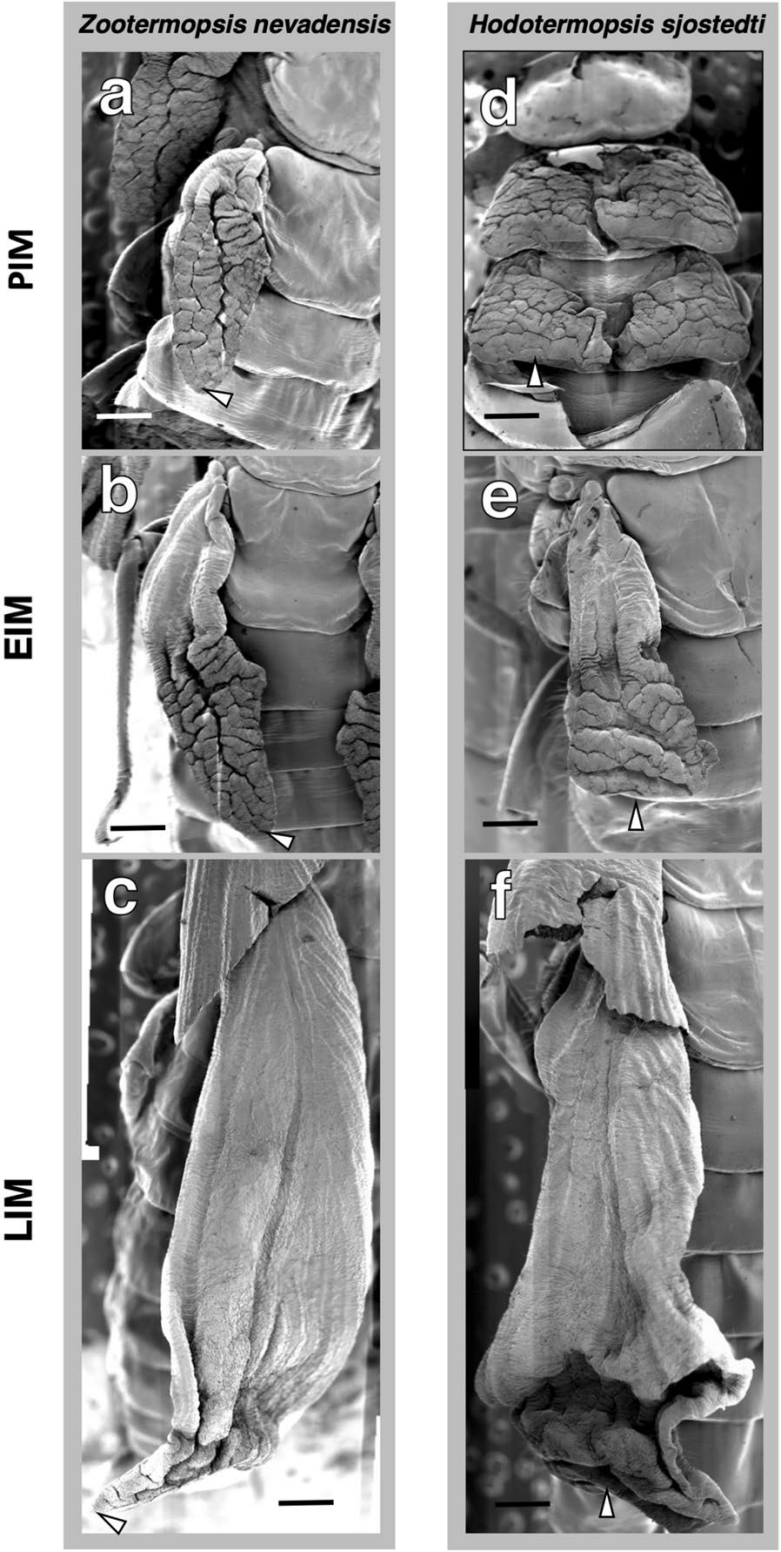
Processes of wing expansion at the imaginal molt in *Z. nevadensis* (**a**–**c**) and *H. sjostedti* (**d**–**f**): PIM (prior to imaginal molt; **a**, **d**), EIM (early stage of imaginal molt; **b**, **e**), and LIM (late stage of imaginal molt; **c**, **f**). Arrowheads indicate the positions of wing tips. All scale bars indicate 500 µm

SEM observations of the thoracic region at the time of imaginal molt showed clear differences in the process of expansion of alate wings between the two species (Fig. 3). In *Z. nevadensis*, the folded structures of wings are only seen inside wing buds, even after the completion of epithelial proliferation just prior to the imaginal molt (Fig. 3a). A major groove is seen in the middle of each folded wing. After the onset of imaginal molt, folded wings started to expand from the basal part (Fig. 3b). Then, at the latest stage of wing expansion, most of the wings were expanded, except that only an apical part of the wing was still folded (Fig. 3c). This process of wing expansion showed that the apical positions of wings were formed at the tips of wing buds.

On the other hand, in *H. sjostedti*, since folded wings could not be contained inside the short wing buds of nymphs, the folded wings extended into the median region of the thoracic part (Fig. 3d), as seen in the section images (Fig. 2o). After the onset of wing expansion, as seen in *Z. nevadensis*, wings were expanded from the basal part, so that the folded shape of the apical part of the wing was maintained (Fig. 3e, f). These results showed that, in the case of *H. sjostedti*, the apical part of the wing did not form at the tip of the wing bud, but rather at a position between the wing-bud tip and the median line of the thorax.

### Expression patterns of wing-patterning genes and hormone-related genes

**Fig. 4:**
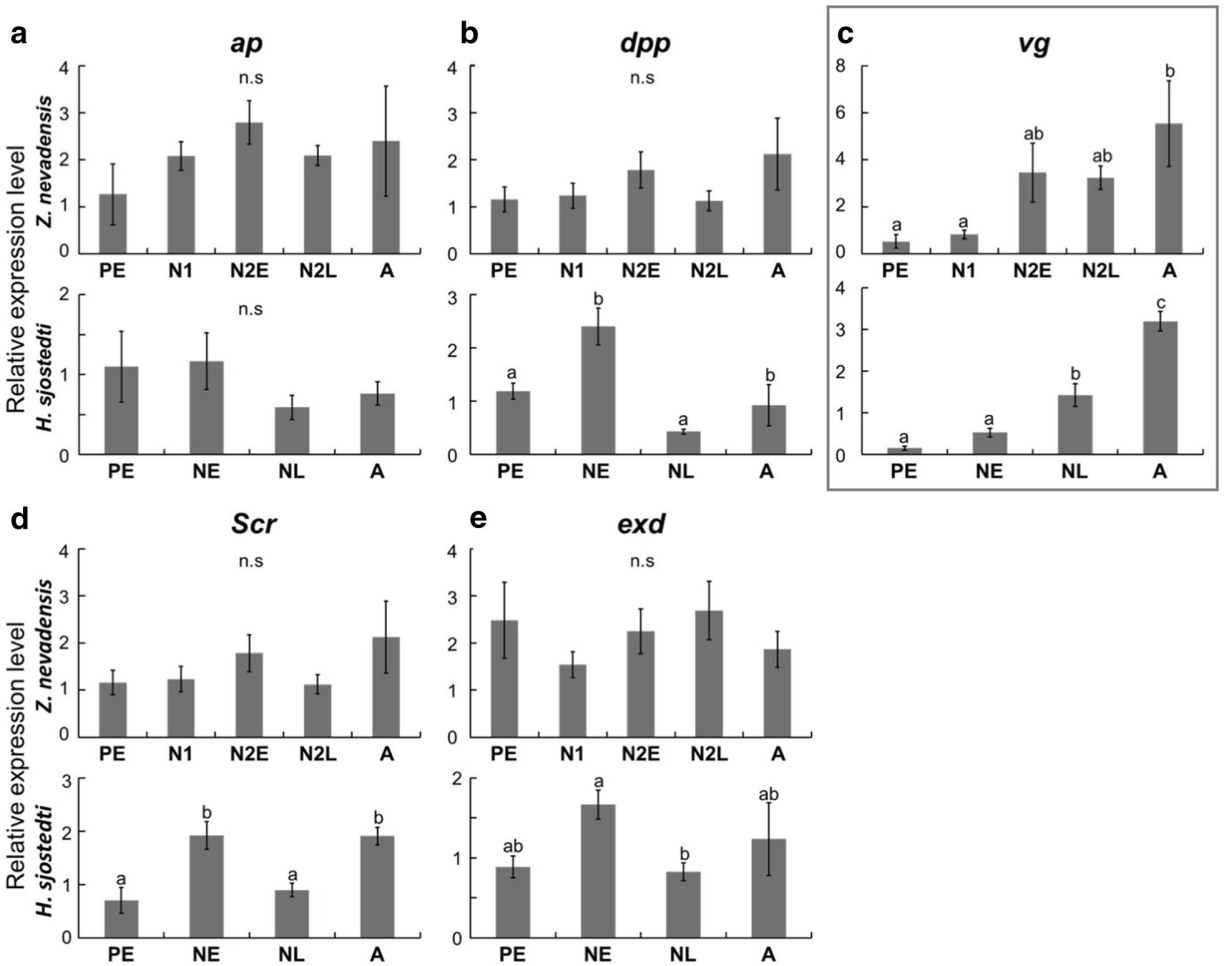
Expression patterns (mean ± SE) of wing-patterning genes, quantified by real-time qRT-PCR; **a** *apterous* (*ap*), **b** *decapentaplegic* (*dpp*), **c** *vestigial* (*vg*), **d** *Sex-combs reduced* (*Scr*), **e** *extradenticle* (*exd*). Different letters on bars indicate significant differences (Turkey’s test, *P* < 0.05). *vg* is boxed to highlight it since it showed the most drastic patterns of stage-specific significant differences that were similar between the two species examined here. The number of samples was *n* = 5 for all the samples of *Z. nevadensis*, and *n* = 3 (PE, A) and *n* = 7 (NE, NL) for *H. sjostedti*

Using real-time qRT-PCR, the expression patterns of wing-patterning genes during nymphal stages were compared between the two species (Fig. 4). In *Z. nevadensis*, significant differences among developmental stages were only detected for the *vestigial* (*vg*) gene (Fig. 4c), although most of the regulatory genes were slightly upregulated at the N2E stage. In *H. sjostedti*, *vg*, *decapentaplegic* (*dpp*), *extradenticle* (*exd*) and *Sex*-*combs reduced* (*Scr*) showed significant differences among developmental stages (Fig. 4b–e; *P *< 0.05, Tukey’s test after one-way ANOVA). *dpp*, *exd* and *Scr* were upregulated at the NE stage, while *vg* was upregulated at the NL stage. Overall, expression peaks were observed at N2E in *Z. nevadensis* and at NE in *H. sjostedti*. In addition, the expression levels in alates (A) were also high in both species. Only *vg* showed a different pattern from other genes, namely, upregulation just before the imaginal molt.

**Fig. 5:**
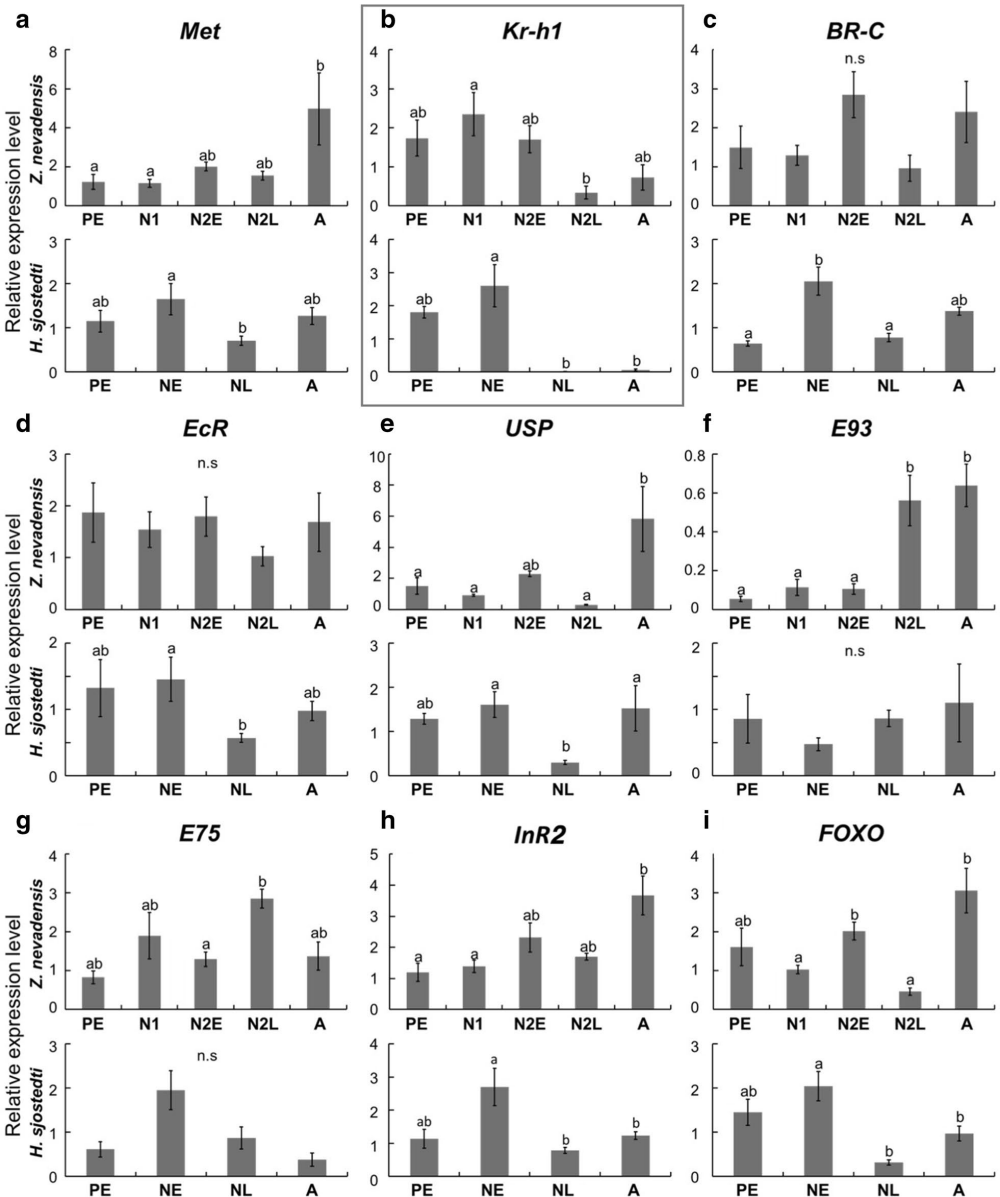
Expression patterns (mean ± SE) of hormone-related genes, quantified by real-time qRT-PCR: **a** *Methoprene*-*tolerant* (*Met*), **b** *Krüppel homolog 1* (*Kr*-*h1*), **c** *Broad-complex* (*BR*-*C*), **d** *Ecdysone Receptor* (*EcR*), **e** *ultraspiracle* (*USP*), **f** *E93*, **g** *E75*, **h** *Insulin Receptor 2* (*InR2*), I) *Forkhead box O* (*FOXO*). Different letters on bars indicate significant differences (Turkey’s test, *P* < 0.05). *Kr*-*h1* is boxed to highlight it since it showed the most drastic patterns of stage-specific significant differences that were shared by the two species examined here. The number of samples was *n* = 5 for all the samples of *Z. nevadensis*, and *n* = 3 (PE, A) and *n* = 7 (NE, NL) for *H. sjostedti*

Hormone-related genes showed more complicated stage-related expression patterns (Fig. 5). Although clear significant differences were not detected, most of the genes examined (*Methoprene*-*tolerant* [*Met*], *Broad*-*complex* [*BR*-*C*], *Ecdysone Receptor* [*EcR*], *ultraspiracle* [*USP*], *Insulin Receptor 2* [*InR2*], *Forkhead box O* [*FOXO*]) showed similar expression patterns to wing-patterning genes, namely expression peaks were seen in N2E in *Z. nevadensis*, and in NE in *H. sjostedti* (Fig. 5a, c–e, h, i). On the other hand, *Krüppel* *homolog 1* (*Kr*-*h1*) showed distinctive patterns in both species; it was clearly downregulated from N2L in *Z. nevadensis*, and from NL in *H. sjostedti* (Fig. 5b). *EcR* and *USP* showed significant downregulation at NL in *H. sjostedti* (Fig. 5d, e). *E93* and *E75* showed significant upregulation at N2L in *Z. nevadensis*, but no significant differences among stages in *H. sjostedti* (Fig. 5f, g). Insulin signaling factors, *InR2* and *FOXO*, showed similar expression patterns in the two species: upregulation at N2E or NE and downregulation at N2L or NL (Fig. 5h, i). Many of the genes (*Met*, *BR*-*C*, *EcR*, *USP*, *E93*, *InR2*, *FOXO*) showed relatively high expression levels at A (Fig. 5a, c–f, h, i).

### Principal component analysis on gene expression patterns

**Fig. 6:**
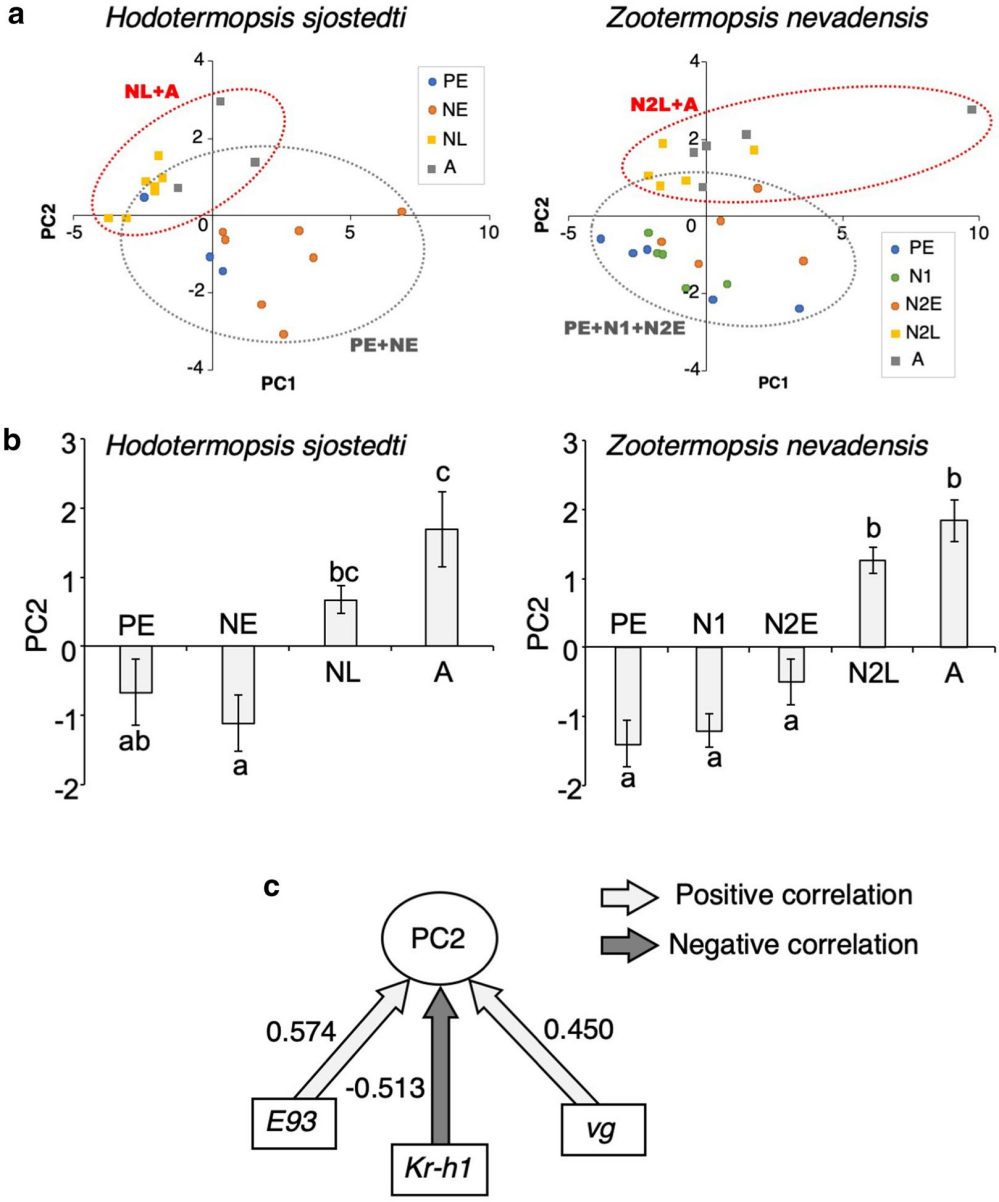
Results of principal component analysis (PCA) based on the gene expression data. **a** Biplots of the principal component scores of examined stages during alate differentiation (PC1 vs. PC2), based on the results of the GLM analysis. The red dotted circles indicate the plots of NL and A in H*. sjostedti* or N2L and A in *Z. nevadensis*. The gray dotted circles indicate the other stages. **b** Principal component scores of PC2 in the respective stages in the two species. Bars indicate standard errors, and alphabetical letters indicate significant differences (*n* = 3–7, Tukey–Kramer test, *P *< 0.05). **c** Schematic model explaining the impacts of expression levels of 3 genes showing higher PC loadings

With all the genes examined in this study, principal component analysis was carried out to compare the overall gene expression patterns between the two species (Fig. 6). To predict which nymphal stage in *Z. nevadensis* corresponds to that in *H. sjostedti*, hypothetical nymphal stage models were made based on the stages in *H. sjostedti*, and they were statistically examined in *Z. nevadensis*. As the results, 4 principal components (PCs) were selected (Additional file 1: Fig. S1), and the cumulative proportion of the 4 PCs was 0.814, that sufficiently explains the variances of the data (Additional file 2: Table S1). Although GLM analysis showed that the PC1 and the PC3 depended on the species difference, the PC2 and the PC4 were shown to be independent from the species difference (Additional file 3: Fig. S2, Additional file 4: Table S2; *n* = 45; Wald test; *P* < 0.05). The PC2 scores increased at NL and A in *H. sjostedti*, the N2L and A in *Z. nevadensis* (Fig. 6a, b; *n* = 3–7; Brown-Forsythe test; *P* > 0.05; one-way ANOVA; *P* < 0.05; Tukey–Kramer test; *P* < 0.05) (Additional file 5: Table S3; Additional file 4: Table S2). In addition, the expressions of *E93*, *Kr*-*h1* and *vg* showed higher contribution ratio to PC2 (Fig. 6c, Additional file 2: Table S1). These indicate that the PC2 well explains the gene expression patterns during the nymphal stages.

## Discussion

### The differences of wing formation

As shown by the results obtained in this study, in *H. sjostedti*, the imaginal molt to alates occurs from the first-instar nymphs with short wing buds. Therefore, the developmental sequence of events of its wing formation differs from that of related species that possess multiple nymphal instars, such as *Z. nevadensis*. Histological and morphological investigations revealed that, in *H. sjostedti*, the extensive epithelial cell proliferation starts during the short-winged nymphal instars so that the complicatedly folded epithelial tissues are compacted all over the thorax (Fig. 2). As a result, the folding and expansion patterns of wings differ from those in *Z. nevadensis* (Fig. 3).

### Heterochronic shifts in wing-patterning and hormone-related genes

From these patterns of epithelial proliferation and wing expansion, it was hypothesized that the expression of genes for wing formation should occur earlier (in short-wing-budded first-instar nymphs) in *H. sjostedti*, as the result of deletion of a nymphal stage. As predicted, this study revealed that many of the genes required for wing formation are upregulated in the second nymphal instar of *Z. nevadensis*, but in the first nymphal instar of *H. sjostedti* (Figs. 4, 5). Interestingly, the pattern of *vg* expression was different from other genes (later in development), probably due to the functional differences among genes. The RNA extraction was carried out from whole thoracic segments, so that these expressions can be responsible not only for the wing development, but also for other tissues including flight muscles, which are also required for alate development. In conclusion, it can be said that heterochronic shifts of wing formation have occurred in the lineage of *H. sjostedti*, so that the developmental events for wing formation that normally happen through two (or more) nymphal instar stages have been compacted within a single nymphal instar.

In endocrine systems in insects, in general, downregulation of the juvenile hormone (JH) pathway is required for the imaginal molt [21]. Therefore, also in termites, a low JH level is known to induce alate differentiation [22–24]. We showed in the present study that the expression level of *Kr*-*h1* was maintained until the early stage of second nymphal instar (N2E) and downregulated at the late stage (N2L) in *Z. nevadensis*, while it was downregulated at the late stage of nymphs (NL) in *H. sjostedti*, suggesting that the imaginal molt is apparently induced during the short-wing-budded nymphal instar in *H. sjostedti*. This situation is similar to the precocious metamorphosis reported in some hemimetabolous insects, in which knockdown of *Kr*-*h1* induced precocious metamorphosis [17, 25, 26]. It is therefore suggested that, during evolution, some adventitious downregulation of JH signaling may have led to a precocious imaginal molt, leading to the lack of second-instar nymphs in *H. sjostedti*.

Among genes that showed no significance in the expression levels, many genes such as *dpp*, *Scr*, *BR*-*C*, *USP*, *E75*, *InR2* and *FOXO*, showed interesting patterns that can explain the heterochronic shifts; NE and NL in *H. sjostedti*, respectively, corresponds to N2E and N2L in *Z. nevadensis*. As for the overall expression patterns, the results of PCA clearly supported the ideas of heterochrony (Fig. 6). All the principal components independent from the species differences supported that N1 and N2E in *Z. nevadensis* corresponds to NE in *H. sjostedti*, and N2L in *Z. nevadensis* corresponds to NL in *H. sjostedti* (Additional file 4: Table S2).

### The developmental constraints of short wing buds

**Fig. 7:**
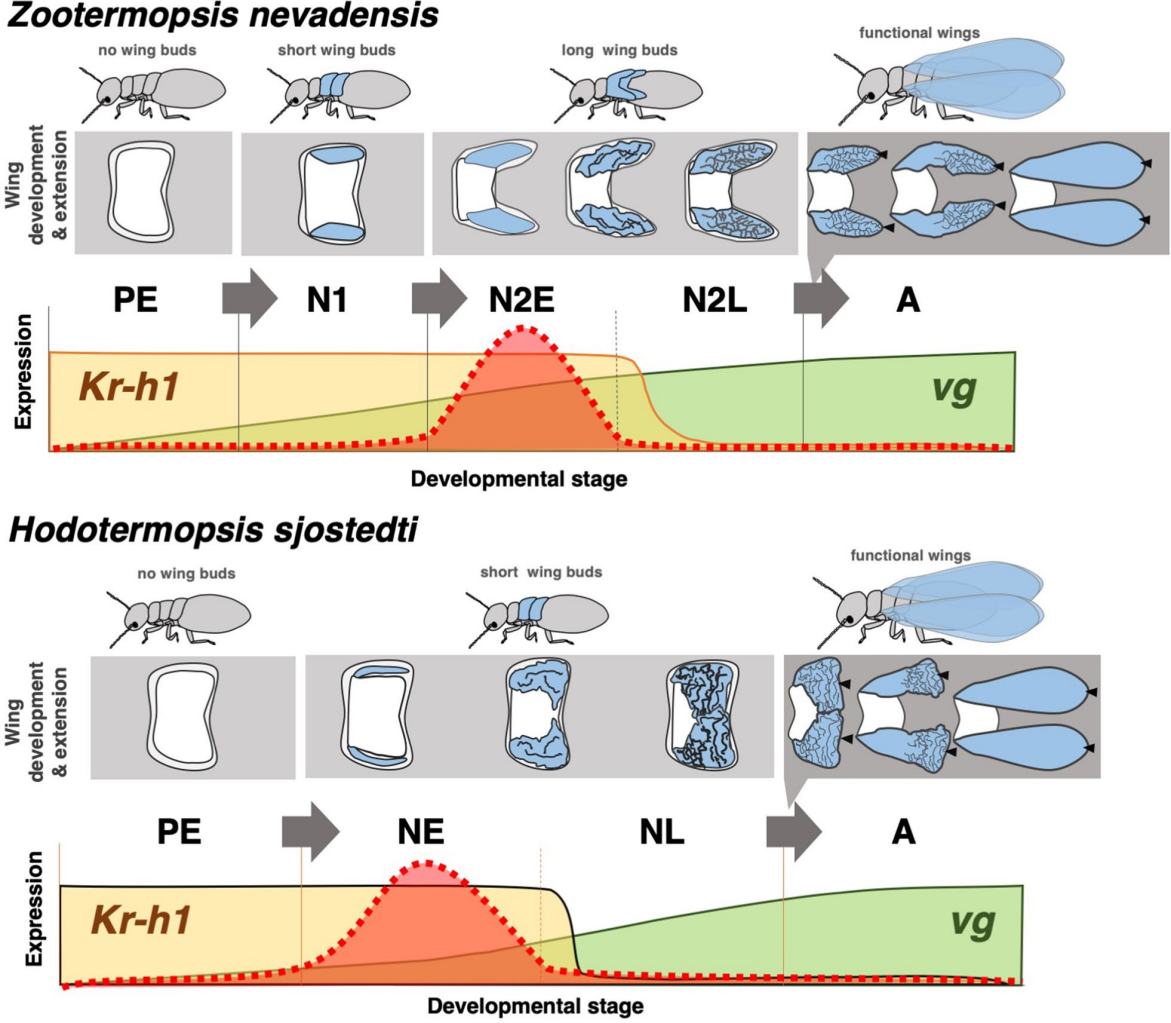
Summary diagrams of wing development and gene expression patterns in *Z. nevadensis* and *H. sjostedti* determined in this study. The processes of wing formation are indicated along the course of nymphal development, followed by the wing expansion at the time of imaginal molt (top row for each species). Expression patterns of representative genes (*vg* and *Kr*-*h1*) are indicated: *Kr*-*h1* is abruptly downregulated at N2L and NL, while *vg* is gradually upregulated at the late stage of the final-instar nymphs. Although statistically significant differences were not detected, expression peaks of most examined genes were seen at N2E in *Z. nevadensis*, while they were seen at NE in *H. sjostedti* (dotted lines)

Also in other species, i.e., *Prorhinotermes inopinatus* and *Termitogeton planus*, a single nymphal instar was reported, although the nymphs possess relatively long wing buds [27, 28]. It is also reported that, in *Nasutitermes princeps*, the number of nymphal instars can change depending on extrinsic factors, inducing short-winged alates called “microimagos” that differentiate from a younger nymphal instar [29]. Although their wings are non-functional, they reproduce as replacement reproductives. However, in the case of *H. sjostedti*, although it has only a single nymphal instar with short wing buds, the alate wings are fully functional. Therefore, this species is suggested to have overcome the developmental constraints caused by the lack of second-instar nymphs (Fig. 7).

### Adaptive significance and intra-colonial conflict

Considering the adaptive significance underlying this unique developmental phenomenon, a candidate factor is thought to be intra-colonial conflict among reproductive candidates. As previously suggested [30], wing bud mutilation could be related to the intra-colonial conflict over reproduction. Mutilated nymphs often undergo regressive molt (molt of a nymph to a pseudergate) in some termite species. In *H. sjostedti*, nymphs whose wing buds were mutilated were often found in relatively large and old colonies, and the regressive molt from nymphs to pseudergates was observed in the laboratory rearing experiments [31]. Such wing bud mutilation could be the selective pressure that led to the reduction of second-instar nymphs in *H. sjostedti*. In some species of *Zootermopsis*, reproductive soldiers or soldier neotenics were reported, and they are suggested to have evolved as a result of intra- or inter-colonial conflicts [32, 33]. In addition, it is also suggested that the caste differentiation pathways in this family (Archotermopsidae) are more plastic than those in other lineages. In this termite family, therefore, these preadaptive conditions, i.e., intragroup conflicts and developmental plasticity, might have facilitated the appearance of soldier neotenics or the heterochronic reduction of nymphal instars.

## Conclusion

In this study, we obtained information about the unique alate differentiation of *H. sjostedti* from the perspectives of histology and gene expression. Our findings suggested that the developmental events for wing formation are compacted into a single nymphal instar in *H. sjostedti*, and as a result, a unique wing formation is seen to compensate for the spatial restriction inside small wing buds, leading to the completion of functional wings. It is suggested that there are behavioral or ecological factors, such as intracolony conflicts among the reproductives, underlying the reduction of nymphal instars. In the future, further studies including behavioral observations will be necessary to get insight into the evolutional process in the reduction of nymphal instars.

## Methods

### Termites

Colonies of *H. sjostedti* Holmgren and *Z. nevadensis* (Hagen) were collected on Yakushima Island, Kagoshima Prefecture, and at Kawanishi City, Hyogo Prefecture, Japan, respectively, in May 2016–2018. *Z. nevadensis* is known to be a species that was introduced to Japan [34]. Nest logs of both species were maintained in the laboratory as stock colonies at approximately 25 °C under constant darkness, as described previously [11, 12, 35]. For their food, moistened pine lumber was occasionally supplied. In *H. sjostedti*, since alates are produced in June under natural conditions, many of the colonies sampled in May contain nymphs. In *Z. nevadensis*, the season for alate production is not so restricted, and the sampled colonies usually contain nymphs throughout most of the year.

### Histological observations

Paraffin sections were made for histological observations on the developmental processes of wings in both species. The examined time points were first-instar nymph (N1) and early and late stage of second-instar nymph (N2E and N2L) in *Z. nevadensis*, and early, mid and late nymphal stage (NE, NM and NL) in *H. sjostedti*. Dissected thoracic parts of nymphs were fixed in FAA fixative (formaldehyde/acetic acid/ethanol = 6:1:16) and preserved in 70% ethanol. Samples were dehydrated in increasing concentrations of ethanol, then transferred into xylene and finally embedded into paraffin. Serial section (8 µm thick) in sagittal and transverse planes was processed routinely and stained with hematoxylin and eosin according to the methods previously reported [36]. Tissues on slides were observed using a BZ-9000 HS All-in-one fluorescence microscope (KEYENCE, Osaka, Japan).

### Scanning electron microscopy

Scanning electron microscopy was carried out to compare the wing bud structures of nymphs and the process of wing expansion during the imaginal molt between *Z. nevadensis* and *H. sjostedti*. This process was classified into 3 time points, i.e., prior to imaginal molt (PIM), early stage of imaginal molt (EIM), and late stage of imaginal molt (LIM). Thoracic parts of individuals before and after the imaginal molt were fixed in FAA. After fixation, the thoracic parts were transferred into increasing concentrations of ethanol followed by *t*-butanol as described in a previous report [12]. In samples at early stages of the imaginal molt, nymphal cuticles were easily removed for observations on newly formed wings. After the transfer into *t*-butanol, the fixed samples were freeze-dried using a Freeze Dryer ES-2030 (Hitachi Global, Tokyo, Japan), and coated with gold ions with an Ion Sputter E-1010 (Hitachi Global, Tokyo, Japan). Samples were observed with a JSM-5510LV scanning electron microscope (JEOL Ltd., Tokyo, Japan).

### Identification of wing-patterning and hormone-related genes

For the comparison of wing development between the two termite species, orthologs of the wing development genes and hormone-related genes were searched in the genome sequence database of *Z. nevadensis* (http://termitegenome.org; [37]) and the transcriptome data of *H. sjostedti* (DDBJ Sequence Read Archive: DRA005483; Sugime et al., [38].). As a result, 5 genes (*ap* [*apterous*], *dpp* [*decapentaplegic*], *vg* [*vestigial*], *Scr* [*Sex*-*combs reduced*], *exd* [*extradenticle*]) were identified as wing development genes and 9 genes (*Met* [*Methoprene*-*tolerant*], *Kr*-*h1* [*Krüppel* *homolog 1*], *BR*-*C* [*Broad*-*Complex*], *EcR* [*Ecdysone Receptor*], *USP* [*ultraspiracle*], *E93*, *E75*, *InR2* [*Insulin Receptor 2*], *FOXO* [*Forkhead box O*]) were identified as hormone-related genes. The protein sequences of those candidate genes in *Drosophila melanogaster* were used as queries for tBLASTn searches against the datasets of the two species. Based on the obtained sequences, primers for real-time qRT-PCR were designed using Primer Express software (ver. 3.0.0, Applied Biosystems, Foster City, CA, USA, Additional file 6: Table S4).

### RNA extraction and real-time qRT-PCR

For the investigations on the expression patterns of genes involved in the wing formation, thoracic parts (prothorax, mesothorax and metathorax excluding guts and legs) were dissected from individuals, and immediately frozen in liquid nitrogen. The examined time points were pseudergate (PE), first-instar nymph (N1), early and late stage of second-instar nymph (N2E and N2L) and alate (A) in *Z. nevadensis*, and pseudergate (PE), early and late nymphal stage (NE and NL) and alate (A) in *H. sjostedti*. Frozen samples were individually preserved in 1.5 ml polypropylene tubes at − 80 °C until RNA extraction. Total RNA was extracted using RNAiso Plus (Takara Bio, Shiga, Japan) according to the manufacturer’s protocol. After the extraction, samples were treated with DNaseI (Thermo Fisher Scientific, Waltham, MA, USA).

For each sample, 900 ng of total RNA was reverse-transcribed with a High Capacity cDNA Reverse Transcription Kit according to the manufacturer’s instructions (Applied Biosystems, Foster City, CA, USA). Quantifications of the relative amounts of target transcripts were then performed using a Fast SYBR Green Master Mix and the sequence detection system ABI PRISM 7500 (Applied Biosystems, Foster City, CA, USA). For identifying an appropriate endogenous control gene, the suitability of different candidate reference genes (*18S rRNA*, *ribosomal protein L13a* [*RPL13a*] and *ribosomal protein 49* [*RP49*] in *Z. nevadensis*; *18S rRNA*, *elongation factor 1 alpha* [*EF1a*], and *ribosomal protein 49* [*RP49*] in *H. sjostedti*) was evaluated using the software geNorm [39] and Normfinder [40]. As a result, *RPL13a* and *RP49* were found to be the most appropriate reference genes for *Z. nevadensis* and *H. sjostedti*, respectively. Data acquisition and analyses were handled with ABI Prism 7500 software ver. 2.0.4 (Applied Biosystems, Foster City, CA, USA), with the relative standard curve method. For statistical analyses to detect significant differences of expression levels, respectively, in each species, Tukey’s multiple comparisons test (*P *< 0.05) was performed after one-way ANOVA (*P *< 0.05), using R 3.5.2 (https://www.r-project.org). The number of samples was *n* = 5 for all the samples of *Z. nevadensis*, and *n* = 3 (PE, A) and *n* = 7 (NE, NL) for *H. sjostedti*.

### Principal component analysis

To examine the correspondence of nymphal stages between the two focal stages based on gene expression patterns, principal component analysis (PCA) was performed. In the analysis, hypothetical models of nymphal stages were made based on the stages of *H. sjostedti*, and were compared with the stages in *Z. nevadensis*. Firstly, the gene expression data that were standardized in the respective species to match the expression ranges among genes, were used for PCA on the correlation matrix to unify the respective gene expression patterns as principal component scores. Thereafter, the respective principal component scores were analyzed by the generalized linear model (GLM) with the Gaussian distribution with identity link. According to the Kaiser’s criterion [41], 4 principal components were selected (Additional file 1: Fig. S1). Species and nymphal stages were used as the explanatory variables. The significance of the respective coefficients was examined by the Wald test. The best model was chosen in accordance with the Akaike’s information criterion (AIC).

## Additional files

**Additional file 1. Fig. S1 ** The scree plot for the principal component analysis. Based on the the Kaiser’s criterion (dotted line), 4 principal components were selected.

**Additional file 2. Table S1.** Cumulative proportions and principal component loadings in the principal component analysis targeting the gene expression levels in Zootermopsis nevadensis and Hodotermopsis sjostedti. Four principal components were selected in accordance with the Kaiser’s rule. Bold letters indicate the value higher than 0.4.

**Additional file 3. Fig. S2.** Biplots of the principal component scores shown in Table S1. The principal components being X or Y axes were selected based on the results of the GLM analysis shown in Table S2. The red dotted circles indicate the plots of NL and A in Hodotermopsis sjostedti or N2L and A in Zootermopsis nevadensis. The gray dotted circles indicate the other stages.

**Additional file 4. Table S2.** Statistical results of the three models hypothesizing the growth stages in *Zootermopsis nevadensis* to match those in *Hodotermopsis sjostedti*. The asterisks indicate the statistical significances of the respective parameters tested by the Wald test (n = 45; P < 0.05). The hypothetical growth stages in the best model independent from species differences are shown as bold. PE, workers; NE, early nymphal stage; NL, late nymphal stage; A, alate.

**Additional file 5. Table S3.** Candidate genes focused in this study.

**Additional file 6. Table S4.** Primer sequences for real time quantitative RT-PCR.
