# Supplementary material for: Reduction of a nymphal instar in a dampwood termite: heterochronic shift in the caste differentiation pathways
Source: EvoDevo. 2019 May 16;10:10. doi: 10.1186/s13227-019-0123-8 (PMC6521406; doi:10.1186/s13227-019-0123-8)
Supplement: Supplementary file 1 — Additional file 1. Fig. S1 The scree plot for the principal component analysis. Based on the the Kaiser’s criterion (dotted line), 4 principal components were selected. [file 13227_2019_123_MOESM1_ESM.pdf]

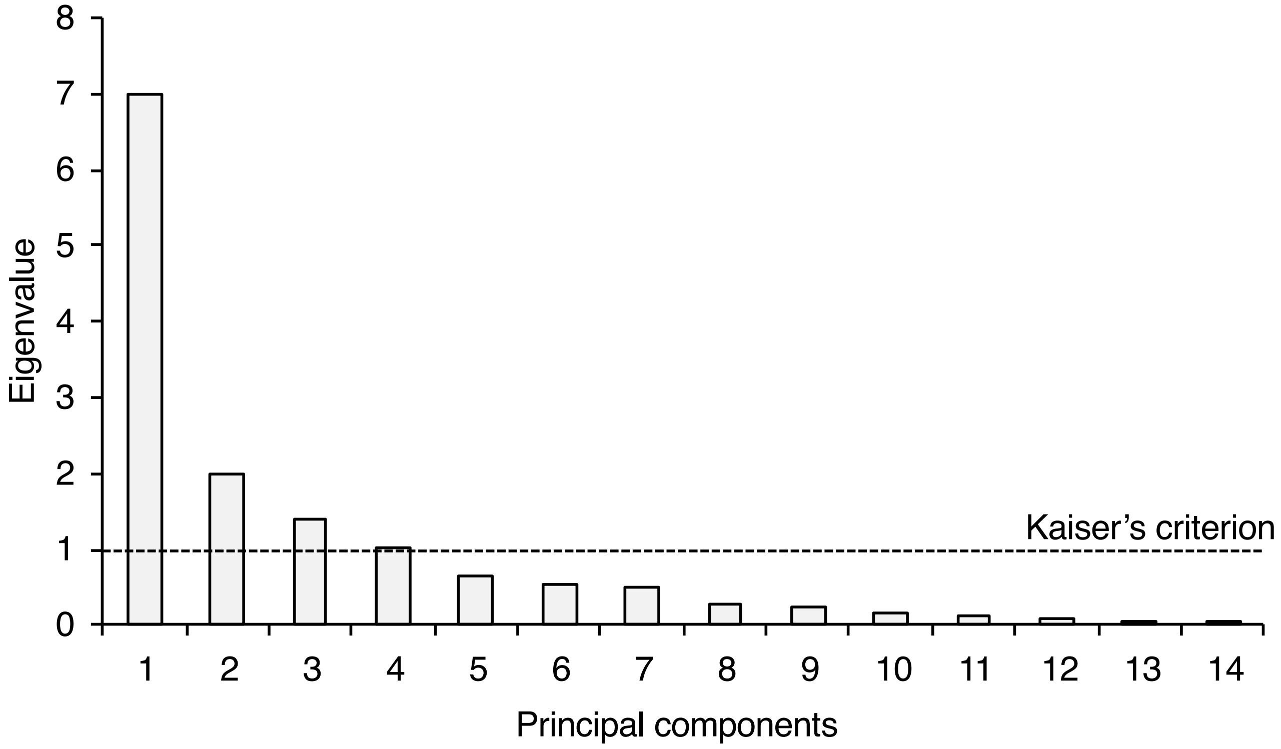

Fig. S1. The scree plot for the principal component analysis. Based on the the Kaiser’s criterion (dotted line), 4 principal components were selected.
