# Supplementary material for: Reduction of a nymphal instar in a dampwood termite: heterochronic shift in the caste differentiation pathways
Source: EvoDevo. 2019 May 16;10:10. doi: 10.1186/s13227-019-0123-8 (PMC6521406; doi:10.1186/s13227-019-0123-8)
Supplement: Supplementary file 3 — Additional file 3. Fig. S2. Biplots of the principal component scores shown in Table S1. The principal components being X or Y axes were selected based on the results of the GLM analysis shown in Table S2. The red dotted circles indicate the plots of NL and A in Hodotermopsis sjostedti or N2L and A in Zootermopsis nevadensis. The gray dotted circles indicate the other stages. [file 13227_2019_123_MOESM3_ESM.pdf]

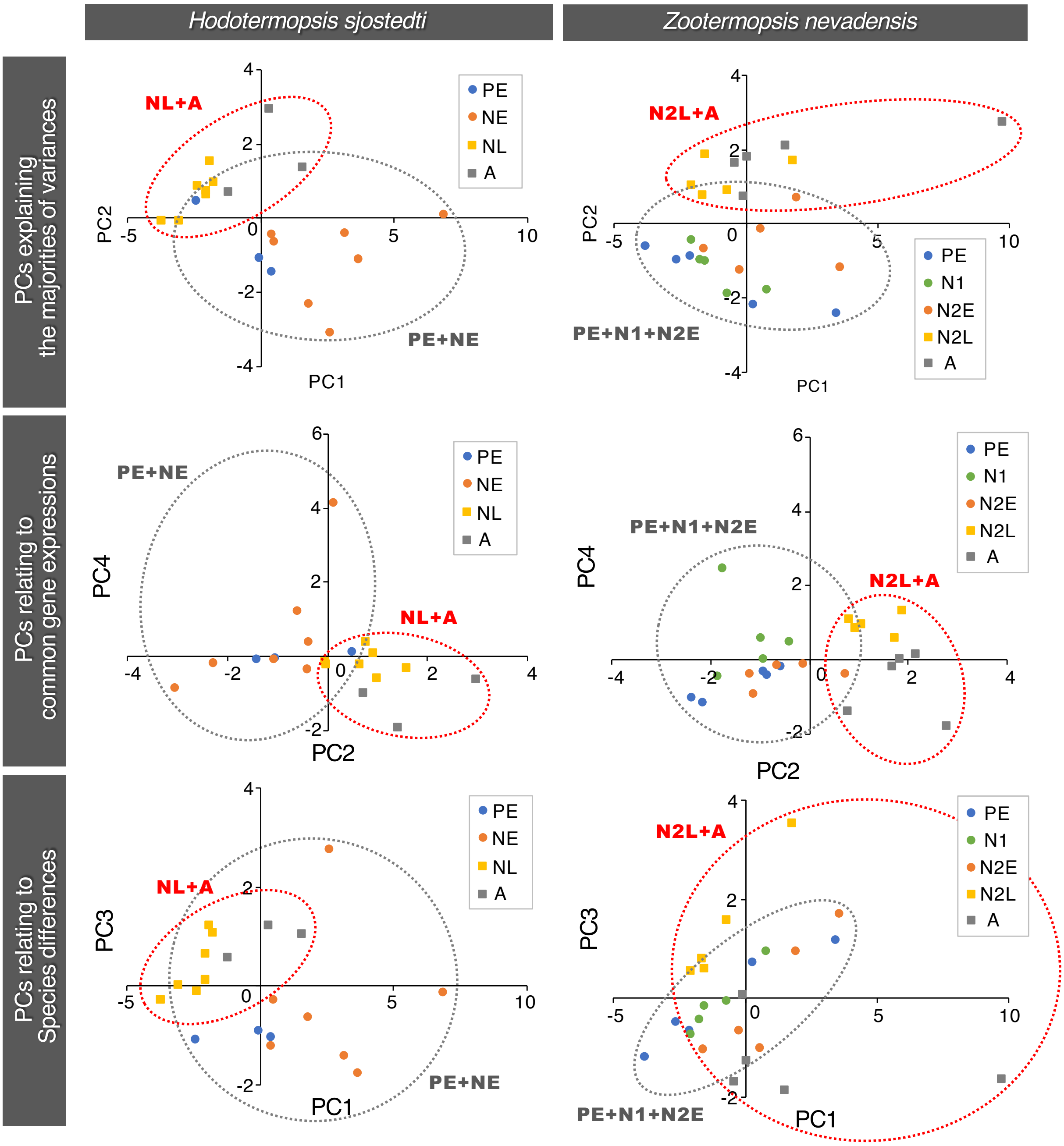

Fig. S2. Biplots of the principal component scores shown in Table S3. The principal components being X or Y axes were selected based on the results of the GLM analysis shown in Table S4. The red dotted circles indicate the plots of NL and A in *H. sjostedti* or N2L and A in *Z. nevadensis*. The gray dotted circles indicate the other stages.
